# Case report of endoprosthesis -Y implantation in severe respiratory failure in the MPSII patient; comparison with literature data

**DOI:** 10.1186/s12890-020-1143-9

**Published:** 2020-04-20

**Authors:** Wojciech Gocyk, Janusz Warmus, Henryk Olechnowicz, Miroslaw Bik-Multanowski, Lukasz Pawlinski, Beata Kiec-Wilk

**Affiliations:** 1 Department of Thoracic Surgery, John Paul II Hospital Krakow, Krakow, Poland; 2 Clinical Department of Medical Genetics, Children University Hospital, Krakow, Poland; 3 Clinical Department of Metabolic Diseases, University Hospital, Krakow, Poland; 4 Department of Metabolic Diseases, Jagiellonian University Medical College, M. Jakubowskiego 2, 30-688 Krakow, Poland

**Keywords:** Mucopolysaccharidosis type II, Respiratory insufficiency, Tracheobronchomalacia, Y-tube stent

## Abstract

**Background:**

The tracheobronchomalacia is a life-threatening complication of mucopolysaccharidosis (MPS) without known effective, optimal treatment. The severe expiratory collapse of the trachea and bronchi is one of causes of the high rate of deaths in the course of airway impairment in MPSII patients.

**Case presentation:**

Due to the adynamic tracheobronchomalacia despite of enzymatic treatment (ERT) in our MPSII patient, a life-saving tracheal bifurcated type-Y endoprosthesis (a self-expanding, metal stent for the prosthesis of tracheal and bronchial stenosis) was implanted. In the followed months, the breathing efficiency improved, but then gradual worsening, progression of bronchi occlusion at the stent border resulted in patient’s death.

**Conclusion:**

The Y-stent implantation appears to be a short-term, life-saving solution without satisfactory long-term effects due to the progress of peripheral bronchomalacia and increased tissue proliferation and granulation, that arises during the illness’ course.

## Background

The treatment for tracheobronchomalacia (TBM) is a challenging problem in mucopolysaccharidosis type II (MPSII) patients [1]. We present the short lasting therapeutic effect ended up with a rapid deterioration of respiratory function, after the tracheal endoprosthesis type-Y implantation in an adult MPSII patient.

## Case presentation

30-year-old MPSII (OMIM: 309900) patient diagnosed in childhood. Due to delayed psycho-motor development, stiffness of joints mainly in the upper limbs and bilateral inguinal hernias. The increased urine glycosaminoglycans (GAGs) excretion and later the hemizygous mutation (c.1035G > T, p.W345C) result confirmed clinical diagnosis. In the course of the underlying disease, the thickening of voice folds and persistent hoarseness without respiratory distress developed. Spirometry examination confirmed a mild restrictive ventilatory defect and a reduced total lung capacity. At the age of 26 an enzyme replacement therapy (ERT, idursulfase) was introduced with a good clinical effect.

**Fig. 1:**
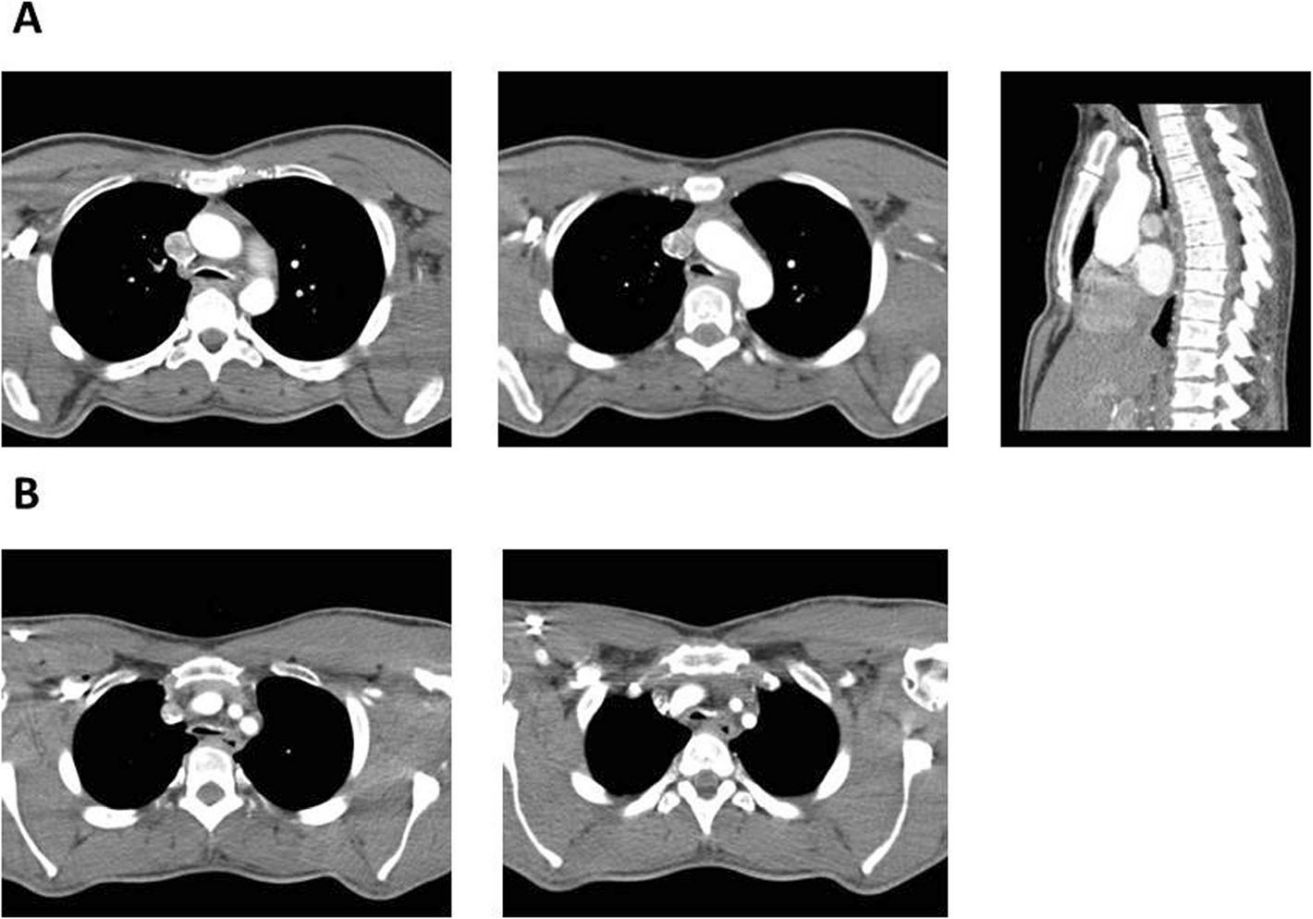
Computed tomography of the respiratory tract in MPSII patient with respiratory insufficiency. The computed tomography (CT) scan performed during the phonation and Valsalva test, revealed: an adynamic tracheobronchomalacia in the lower parts of the respiratory tract with a deformation and significant reduction of the respiratory pathway diameters. The deformed trachea had reduced sagittal dimension with a reduction of lumen in a distance of at least few centimetres, at its narrowest point to 0,4cm^2^, 4,5 × 9 mm. **a**) A significant reduction in the airway lumen observed in the MPS patient - multilevel tracheomalacia with excessive collapsibility of the trachea. **b**) Bronchomalacia with a significant, multilevel reduction in the peripheral airway lumen

During the fourth year of ERT, after a viral infection of the respiratory tract, the patient reported cough and exercise-related dyspnea. A phoniatric examination revealed swallowed mucosa and a polyp structure on the right vocal fold. Due to the inflammatory aetiology, steroidotherapy (methylprednisolone) was commenced with a long-acting, selective, H1-receptor antagonist (bilastine); resulting in a clinical improvement. After 4 months the symptoms recurrence with a worsening of the spirometry tests results occurred. The patient suffered from shortness of breath and attacks of unproductive, night-time cough. The computed tomography scan performed during the phonation and Valsalva test, revealed: a concentric soft tissue hypertrophy of the upper and middle laryngeal part with a reduction of lumen diameter -stating an adynamic tracheobronchomalacia. (Fig. 1). Laboratory tests confirmed a partially compensated respiratory acidosis (pH: 7.310 [7.350–7.450], pCO_2_: 56.5 mmHg [35–48], pO_2_: 67.6 mmHg [83–108], HCO3act: 29 mmol/l [22–28], BE(B) c (ABE): 2.9 mmol/l [− 2.0–3.0], sO_2_: 75.9% [95.0–99.0]). Patient refused continuous positive airway pressure (CPAP) therapy.

Due to the deteriorating condition despite conservative treatment, patient was qualified for the bifurcated tracheobronchial, Y-stent implantation. The procedure of housing the stent was complicated by the larynx and lower airways oedema, and required tracheostomy. The self-expanding, metal stent for the prosthesis of tracheal and bronchial stenoses was placed through the tracheostomy opening, producing a combination of tracheostomy and bifurcated-tracheobronchial stent.

**Fig. 2:**
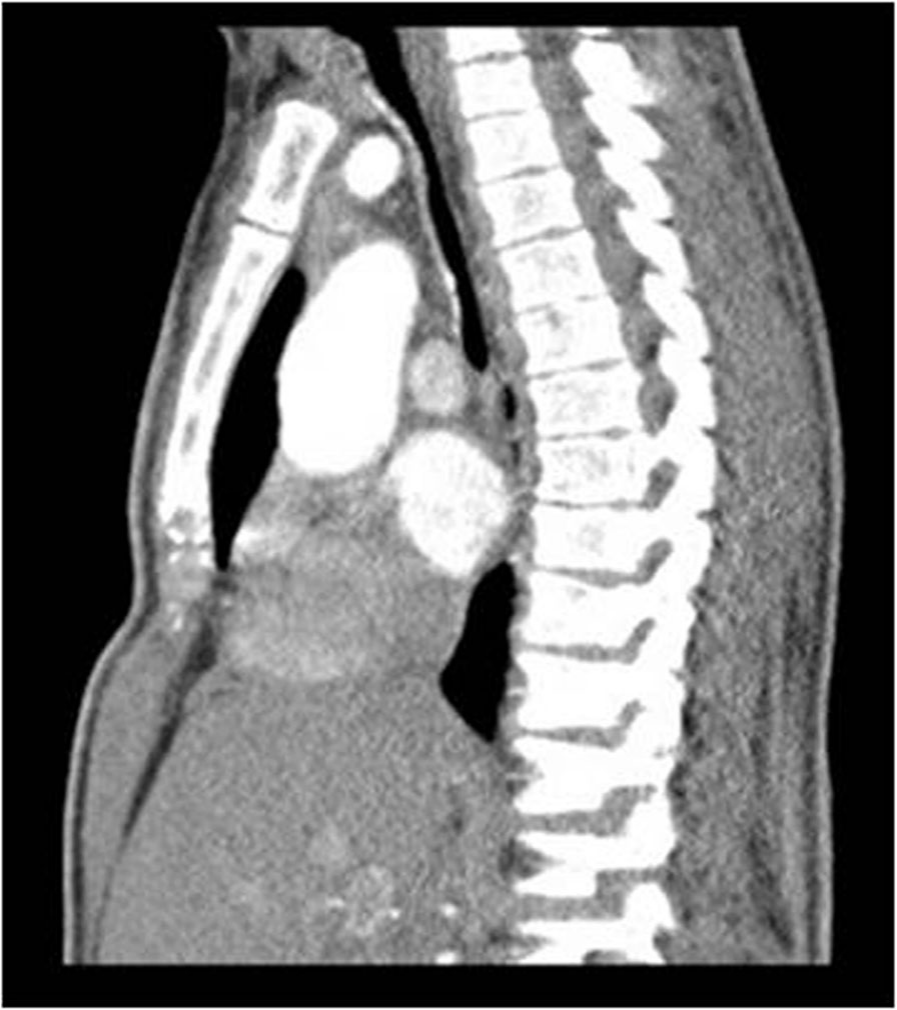
Computed tomography (CT) of the respiratory tract in MPSII patient after bifurcated-tracheobronchial stent implantation. The CT scan confirmed the correct Y-stent position and the amelioration in the dimensions of the airways at the level of the trachea and major bronchi

The patient’s breath efficiency significantly improved: pH: 7.385, pCO_2_: 43.5 mmHg, pO_2_: 84.7 mmHg, HCO3act: 26.4 mmol/l, BE: 0.4 mmol/l, sO_2_: 95.4%. Imaging studies confirmed the effective dilation of patient’s airways (Fig. 2). Unfortunately 3 weeks later, patient’s status deteriorated. The new stenosis of the major bronchus, caused by tissue granulation and bronchomalacia distally from the prosthesis, was stated. The granular material was coagulated using argon plasma, resulting with the clinical improvement. The ERT, once a week, was continued during the whole time, as well as respiratory regimen at home (nebulizers, aspirations).

Two months after leaving the hospital, during the tracheostomy tube toilet, patient suffered from severe dyspnea (sO_2_ < 60%). The unconscious patient was transported to a district hospital. Two episodes of a cardiac arrest followed by asystole appeared, with subsequent effective resuscitation procedures. After a third cardiac arrest in pulseless-electrical-activity mechanism and non-effective CRP the patient’s death was declared.

## Discussion and conclusions

The guidelines do not give clear recommendations how to treat a severe respiratory failure in MPSII patient [2]. The article describes the sudden respiratory decompensation in a MPS patient due to adynamic TBM followed by a severe tissue granulation at the border of implanted respiratory Y-stent.

The limitation of this report is the fact that it is the single-centre observation however, it presents a case where the rare occurrence of the disease (1:100 up to 150,000 male births) per se, limits the clinical experience of the centres, especially when it refers to the adult MPSII patients.

The severe expiratory collapse of the trachea and bronchi can explain the high rate of deaths caused by airway impairment in MPSII patients [3, 4].

The severe obstructive respiratory symptoms develop mainly in MPS I, II and VI [1, 5–7]. It is known that ERT is more effective in preventing the upper airway obstruction, penetrating to the bronchi soft tissue but not to the trachea cartilaginous skeleton [6] thus does not prevent the bronchomalacia [6].

The TBM causes difficulties during intubation and stent implantation and may require external access to the airways. Despite a relatively short-term improvement; stent implantation may even accelerate the sequence of events that finally lead to a patient’s death. The increased tissue granulation and mucus production, causing the airway obstruction, observed on the Y-stent border, could have resulted from the irritating role of the prosthesis on the mucosa respiratory tissues. A similar phenomenon was observed in the implanted coronary stent, leading to the restenosis [8]. Our article draws attention to two problems: the severe morbidity (an acute complication during intubation) [9, 10] and the mortality of MPSII patients after a Y-stent implantation. The positive short-term effect after airway stenting in MPS II and VI patients, with high long-term morbidity, was reported [11, 12]. Despite this, authors suggest that in case of TM with severe tracheal stenosis the stent implementation should be mandatory, life-saving procedure [12]. The main ‘take-away’ lesson of this case report are the weak sides of this type of therapy, but until now we do not have the better way of adynamic TBM and respiratory decompensation treatment in MPS.

Clinical reports suggest the advantages of CPAP therapy in respiratory decompensation in MPS [12, 13]. But such therapy will be most effective in the dynamic TBM and there is still no optimal model for the treatment of its adynamic form.

## Data Availability

The data that support this case report are not publicly available. Data may be however available from the corresponding author upon reasonable request and with permission of patient’s family member.

## Abbreviations

CPAP: continuous positive airway pressure
ERT: enzyme replacement therapy
MPS II: mucopolysaccharidosis
TBM: tracheobronchomalacia
